# Chromosomal distribution of pTa-535, pTa-86, pTa-713, 35S rDNA repetitive sequences in interspecific hexaploid hybrids of common wheat (*Triticum aestivum* L.) and spelt (*Triticum spelta* L.)

**DOI:** 10.1371/journal.pone.0192862

**Published:** 2018-02-15

**Authors:** Klaudia Goriewa-Duba, Adrian Duba, Michał Kwiatek, Halina Wiśniewska, Urszula Wachowska, Marian Wiwart

**Affiliations:** 1 Department of Plant Breeding and Seed Production, University of Warmia and Mazury in Olsztyn, Olsztyn, Warmian-Masurian Voivodeship, Poland; 2 Department of Entomology, Phytopathology and Molecular Diagnostics, University of Warmia and Mazury in Olsztyn, Olsztyn, Warmian-Masurian Voivodeship, Poland; 3 Institute of Plant Genetics, Polish Academy of Sciences, Poznań, Wielkopolskie Voivodeship, Poland; 4 Department of Genetics and Plant Breeding, Poznań University of Life Sciences, Poznań, Wielkopolskie Voivodeship, Poland; Huazhong University of Science and Technology, CHINA

## Abstract

Fluorescent *in situ* hybridization (FISH) relies on fluorescent-labeled probes to detect specific DNA sequences in the genome, and it is widely used in cytogenetic analyses. The aim of this study was to determine the karyotype of *T*. *aestivum* and *T*. *spelta* hybrids and their parental components (three common wheat cultivars and five spelt breeding lines), to identify chromosomal aberrations in the evaluated wheat lines, and to analyze the distribution of polymorphisms of repetitive sequences in the examined hybrids. The FISH procedure was carried out with four DNA clones, pTa-86, pTa-535, pTa-713 and 35S rDNA used as probes. The observed polymorphisms between the investigated lines of common wheat, spelt and their hybrids was relatively low. However, differences were observed in the distribution of repetitive sequences on chromosomes 4A, 6A, 1B and 6B in selected hybrid genomes. The polymorphisms observed in common wheat and spelt hybrids carry valuable information for wheat breeders. The results of our study are also a valuable source of knowledge about genome organization and diversification in common wheat, spelt and their hybrids. The relevant information is essential for common wheat breeders, and it can contribute to breeding programs aimed at biodiversity preservation.

## Introduction

The preservation of biodiversity in living organisms, including major crop species, is one of the key challenges in the 21^st^ century. The aim of modern breeding programs is to produce high-yielding cultivars. Over the years, the above has considerably narrowed down genetic pools of many crop species, including common wheat (*Triticum aestivum* L). In contemporary wheat cultivars, reduced genetic variation increases susceptibility to environmental stressors. Wild emmer wheat, *Triticum dicoccoides* (Körn. ex Asch. & Graebn.) Schweinf. [1], is the donor of A and B genomes to contemporary tetraploid wheats. It is believed that the A genome originates from diploid wheat *Triticum urartu* Thum. ex Gandil, and the B genome—from the species *Aegilops speltoides* Tausch. [2]. Mutations in the above genomes and variations resulting from crosses with related taxa have led to the emergence of new species such as durum wheat (*Triticum durum* Desf.), Polish wheat (*Triticum polonicum* L.) and Khorasan wheat (*Tritcum turanicum* Jakubz.). Hexaploid wheats carry the third genome, D, which was introduced when wheat the AABB amphiploid was crossed with *Aegilops tauschii* Coss. (= *Triticum tauschi* Coss.) with the DD genome [3]. Common wheat and spelt (*Triticum spelta* L.) belong to the above group [4,5]. Genetic drift and natural and artificial selection have led to the emergence of local varieties that were very well adapted to specific environmental conditions. Today, the above varieties are of low economic significance, and they have been replaced by high-yielding cultivars that account for more than 90% of global wheat production [6]. These cultivars have been developed by crossing a relatively small number of local varieties and cultivars. In consequence, they are characterized by low genetic variation and a relatively high degree of relatedness [5].

In the past two decades, consumers have shown a growing interest in high-quality food products. Due to a steady decrease in the genetic variation of common wheat and lower nutritional value of wheat grain proteins in comparison with other cereals, breeders increasingly often rely on species closely related to *T*. *aestivum* to produce cultivars with improved nutritional value [7,8]. The growing popularity of spelt can be attributed to its relatively low agronomic requirements and high resistance to abiotic and biotic stress. Spelt is characterized by hulled grains and genetic polymorphism, which effectively prevent the spread of pathogenic infections [9,10]. Wiwart et al. [11] demonstrated that spelt is more resistant to *Fusarium culmorum* infections than common wheat. Today, spelt is produced mainly in organic farming systems [10]. Growing levels of consumer awareness contribute to the popularity of spelt. According to Waga et al. [12] and Escarnot et al. [13], spelt is a valuable source of genes responsible for high nutritional value and a high protein content of grain. As a result, spelt grain is characterized by high concentrations of essential amino acids, including tyrosine, leucine and isoleucine, as well as valuable nutrients such as zinc, magnesium and iron ions. According to Waga et al. [12], spelt and common wheat hybrids are characterized by similar nutritional value. There is evidence to indicate that spelt delivers health benefits and can be used in the production of hypoallergenic foods [14,15]. However, the non-allergenic properties of spelt were not confirmed by Waga et al. [12], Ruibal-Mendieta et al. [16] or Pahr et al. [17]. Spelt grain proteins have higher nutritional value than common wheat proteins, and the protein content of spelt can exceed 16.5% of dry weight [8]. The quality and nutritional value of *T*. *spelta* grain is superior to that *T*. *aestivum*. Common wheat grain is characterized by free threshability, considerable resistance to lodging and high yield potential, which prompts breeders to develop hybrids of these two closely related species. Research into common wheat and spelt hybrids revealed that the expression of heterosis in F_1_ individuals can reach 40%. The grain of common wheat and spelt hybrids was also characterized by higher nutritional value and processing suitability than the grain of parental forms [18]. Studies investigating hybrids’ resistance to selected pathogens have demonstrated that progeny resistant to brown rust can be produced if one of the parental components is resistant to this pathogen [19]. Common wheat and spelt hybrids are characterized by new agriculturally useful traits, in particular high nutritional value and processing suitability of grain [18].

*T*. *aestivum* and *T*. *spelta* hybrids have never been subjected to cytogenetic analyses. The aim of this study was to: (1) describe the karyotype of *T*. *aestivum* and *T*. *spelta* hybrids and their parental components (three common wheat cultivars and five spelt breeding lines), (2) to identify chromosomal aberrations in the analyzed genomes, and (3) to analyze the distribution of polymorphism of repetitive sequences in the examined hybrids.

## Materials

The experimental material comprised the grain of F_7_ hybrids from single crosses between *T*. *spelta* x *T*. *aestivum* and *T*. *aestivum x T*. *spelta* and their parental forms: spring spelt breeding lines (denoted S10, S11, S12, S13 and S14), selected at the Department of Plant Breeding and Seed Production of the University of Warmia and Mazury in Olsztyn, Poland, and three cultivars of common wheat: Torka, Kontesa and Zebra (Table 1, S1 Table). The field experiment was performed at the Agricultural Experiment Station in Bałcyny, Poland (53°36'N, 19°51'E). Spelt and wheat were grown and harvested in accordance with good agricultural practice standards. Parental forms and their hybrids were subjected to cytogenetic analyses to determine their karyotypes, to localize and identify chromosomal aberrations, and to develop a physical map of repetitive sequences.

**Table 1:** The analyzed hybrids and their parental forms.

No	Hybrid	No	Hybrid	No	Hybrid	No	Parent
1	TORKA x S10	9	KONTESA x S14	17	S12 x TORKA	25	TORKA
2	TORKA x S11	10	ZEBRA x S10	18	S13 x TORKA	26	KONTESA
3	TORKA x S12	11	ZEBRA x S11	19	S14 x TORKA	27	ZEBRA
4	TORKA x S14	12	ZEBRA x S12	20	S10 x KONTESA	28	S10
5	KONTESA x S10	13	ZEBRA x S13	21	S11 x KONTESA	29	S11
6	KONTESA x S11	14	ZEBRA x S14	22	S12 x KONTESA	30	S12
7	KONTESA x S12	15	S10 x TORKA	23	S13 x KONTESA	31	S13
8	KONTESA x S13	16	S11 x TORKA	24	S14 x KONTESA	32	S14

## Methods

### DNA isolation

The leaves of three-week-old seedlings of spelt, common wheat and wheat hybrids were collected. DNA was isolated with a ready-to-use Genomic Micro AX Plant Gravity Kit (A&A Biotechnology, Poland). The quantity and quality of DNA was determined with a spectrophotometer (nanoMaestro Gen, Poland) at 260 nm and 280 nm wavelength. Extracted DNA was additionally purified before further analysis with the use of the Anti-Inhibitor Kit (A&A Biotechnology, Poland). The 1BL/1RS translocation in investigated parental components and common wheat-spelt hybrids was identified by PCR according to the method described by Iqbal and Rayburn [20] with specific primers JO71F1 5’-TAAGCCGTAAAGCATGGTGCAC-3’ and J07IR1 5’-CTTCAACGAAAT GTT TTC CTC TTC-3’. Total reaction volume was 20 μl.

### Preparation of chromosome spreads

Seed germination and the accumulation of chromosomes during metaphase division in embryonic wheat roots were determined according to the method described by Kwiatek et al. [21]. Mitotic chromosome preparations were obtained from root tips digested in an enzyme mixture: 20% (v/v) pectinase (Sigma), 1% (w/v) cellulase (Calbiochem) and 1% (w/v) Onozuka R-10 cellulase (Serva) diluted in 0.01M sodium citric buffer (pH 4.8). Root tips were macerated for at least 150 minutes at 37°C. After maceration, the enzyme mixture was removed, and roots were rinsed with sodium citric buffer at room temperature. Root tips were placed on a slide in a drop of ice-cold 60% acetic acid and dispersed with a metal needle. The dispersed material was covered with a coverslip and pressed down. Slide quality was verified by phase-contrast microscopy.

### DNA probes

DNA probes three repetitive sequences, pTa-86 (GenBank accession number KC290896.1), pTa-535 (KC290894.1) and pTa-713 (KC290900.1), were amplified from the clones listed in the BAC library of wheat developed by Komuro et al. [22]. Additional repetitive sequence, 35S rDNA (KC290907) was also used in this study [23]. Specific primers were designed in the Primer3 program [24] to amplify selected sequences (S2 Table). Primer properties were verified with OligoCalc [25]. PCR conditions were as follows: 95°C for 5 minutes, 35 cycles of 95°C for 30 seconds, annealing temperature appropriate for each primer pair (pTa-86: 58.5°C, pTa-535: 58°C, pTa-713: 59°C, 35S rDNA: 59°C) for 30 seconds, 72°C for 90 seconds and 72°C for 5 minutes. All sequences were labeled with the nick-translation kit (Sigma) according to the manufacturer’s instructions. Probe pTa-86 was labeled with digoxigenin-11-dUTP (Roche), probe pTa-535 –with tetramethyl-5-dUTP-rhodamine (Roche) and probe pTa-713 –with Atto 647 (Jena BioScience). 35S rDNA probe was labeled with Atto 647 (Jena BioScience).

### Fluorescent *in situ* hybridization

The FISH procedure was carried out according to the protocol described by Kwiatek et al. [21]. Chromosomes were treated with RNase (100 μg per milliliter) and incubated in a moist chamber for 60 minutes at a temperature of 37°C. After incubation, the samples were twice rinsed in 2x SSC for 5 minutes. They were deproteinized in formaldehyde (4%) diluted with 1x PBS for 15 minutes at room temperature. Deproteinized samples were twice rinsed in 2x SSC for 5 minutes and dehydrated in a graded series of alcohols: 70%, 90% and 100%. Chromosomal DNA in the presence of hybridization mixture (50% formamide, 10% dextran sulfate, 20x SSC, 0,1% SDS, 30 μg of SHS, 70 ng of probes and water) was denatured at 75°C for 10 minutes in under cover slip and stabilized on ice. Drops of the hybridization mixture were applied to glass slides, the specimens were incubated overnight in a moist chamber at 37°C. The next day specimens were rinsed in 2x SSC, 0,1x SSC, 0,1x SSC and 2x SSC solutions in a water bath at a temperature of 42°C, followed by 2x SSC at room temperature. Before the application of antibody solutions, the specimens were additionally rinsed in 4x SSC+0.2% Tween 20 at room temperature. 20 μg per milliliter of anti-digoxigenin-fluorescein antibody (Roche) was applied, then the specimens were incubated in a moist chamber for 60 minutes to increase signal intensity and were rinsed in 4x SSC+0.2% Tween 20 heated to 37°C and in 2x SSC at room temperature. The specimens were dehydrated in a graded series of alcohols: 70%, 90% and 100%, and were mounted with DAPI and then with Vectashield. Image processing was carried out using Olympus Cell-F (version 3.1; Olympus Soft Imaging Solutions GmbH: Münster, Germany) imaging software and Photoshop CS3 software (version 10.0.1; Adobe Systems, USA). After documentation of the FISH sites, the slides were washed as described in Probe elution section and dried and used for second FISH experiment. In the first FISH trial, three probes were applied: pTa-86, pTa-535 and pTa-713. After elution, probes pTa-86 and pTa-535 were also applied (in order to identify the particular chromosomes) and third probe pTa-713 was added. The identification of particular chromosomes were made by comparing the signal patterns of tested probes hybridized to hexaploid wheat according to Komuro et al. [22] and Kwiatek et al. [21].

### Probe elution

After documentation of the FISH sites, the analyzed slides were washed (2×60 min in 4×SSC Tween, 2×5min in 2×SSC, at room temperature) and after alcohol washes were dried and used for second FISH experiment. They were dehydrated in a graded series of alcohols: 70%, 90% and 100%.

## Results

### Karyotyping

**Fig 1:**
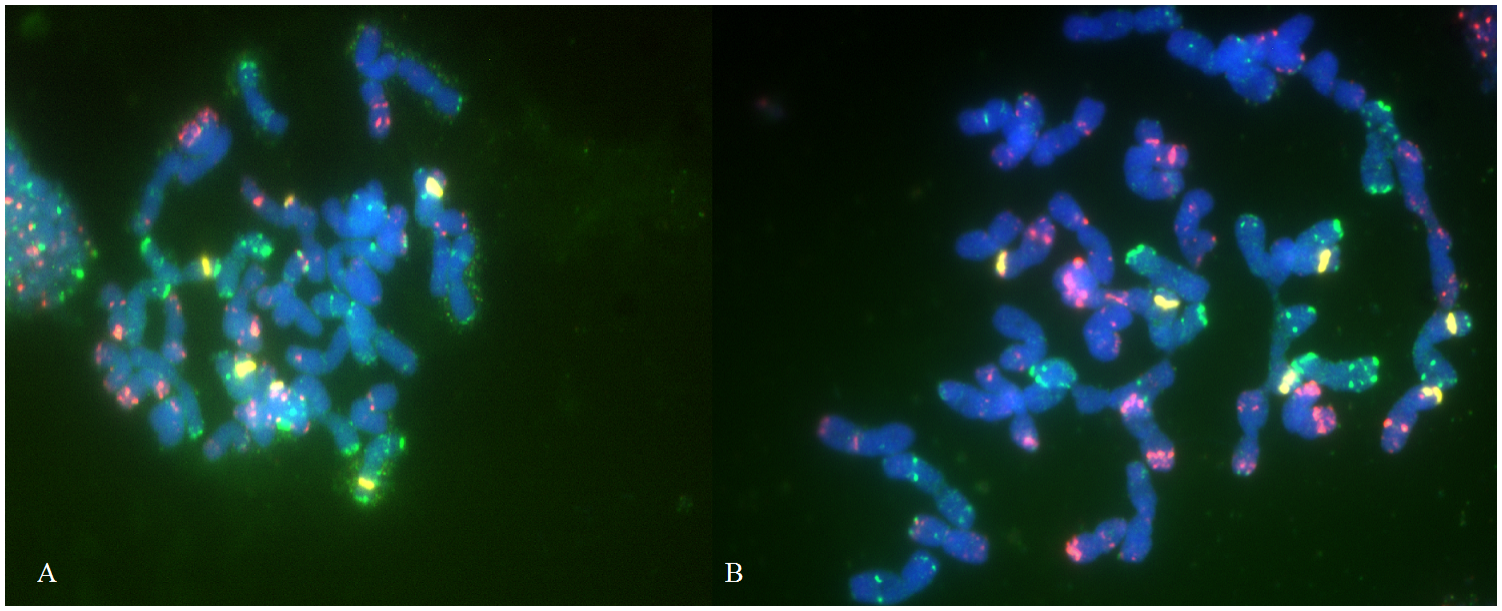
A cluster of chromosomes in spelt line (S14) (A) and separated chromosomes of common wheat cultivar Zebra (B).

Parental forms (*T*. *spelta* and *T*. *aestivum*) and their simple-cross hybrids were characterized by similar genome composition (BBAADD, 2n = 6x = 42). Unlike in common wheat accessions, the chromosomes in spelt lines bred by the authors were difficult to separate due to high cytoplasm density (Fig 1).

The FISH method enabled the identification of chromosome structure and possible aberrations. Structural aberrations such as translocations, inversions and deletions were not observed in parental wheat cultivars (*T*. *spelta* breeding lines S10-S14 and *T*. *aestivum* cultivars Torka, Kontesa and Zebra). In our experiment, attempts were also made to identify the 1BL/1RS translocation, however, it was not detected in any of the progenitors or their hybrids. PCR-based identification of 1BL/1RS translocation also showed the absence of 1BL/1RS translocation.

### Physical mapping of repetitive sequences

**Fig 2:**
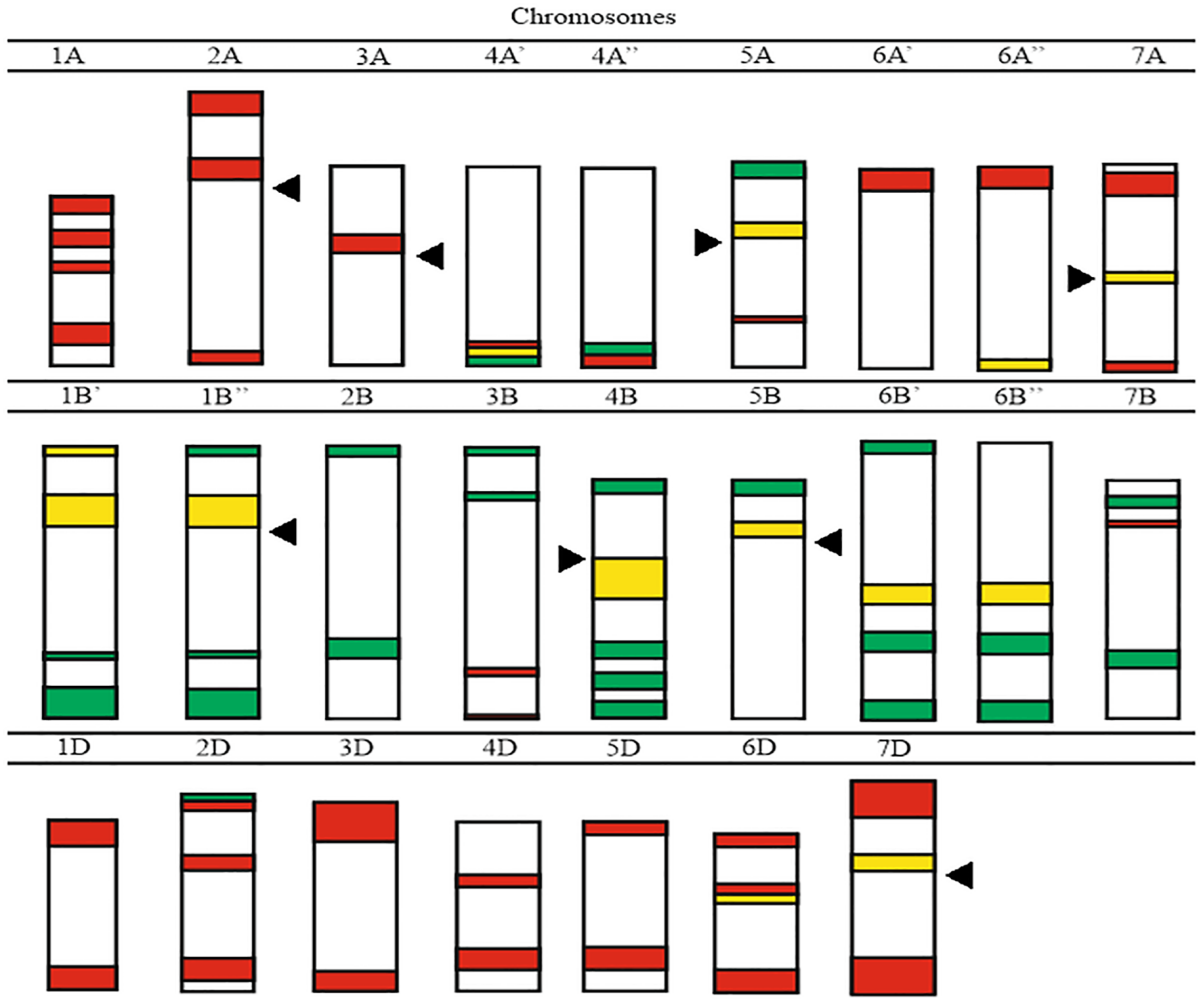
A physical map of repetitive sequences pTa-535 (red signals), pTa-86 (green) and pTa-713 (yellow) in analyzed lines of common wheat and spelt and their hybrids. Arrowheads indicate centromere positions. In the absence of an arrowhead, a chromosome is considered metacentric.

The FISH method was also used to analyze the distribution of repetitive sequences in wheat chromosomes. A-, B- and D-genome chromosomes were identified by comparing the labeling patterns developed by Komuro et al. [22]. In our study, the distribution analysis of pTa-535, pTa-86 and pTa-713 and 35S rDNA repetitive sequences in the chromosomes of common wheat and spelt lines and their hybrids (Fig 2) revealed considerable similarity with selected polymorphic sites. The long arm of a chromosome is termed the L arm and the short arm is indicated by S letter. Both indicators are located next to particular chromosome number.

### A-genome chromosomes

**Fig 3:**
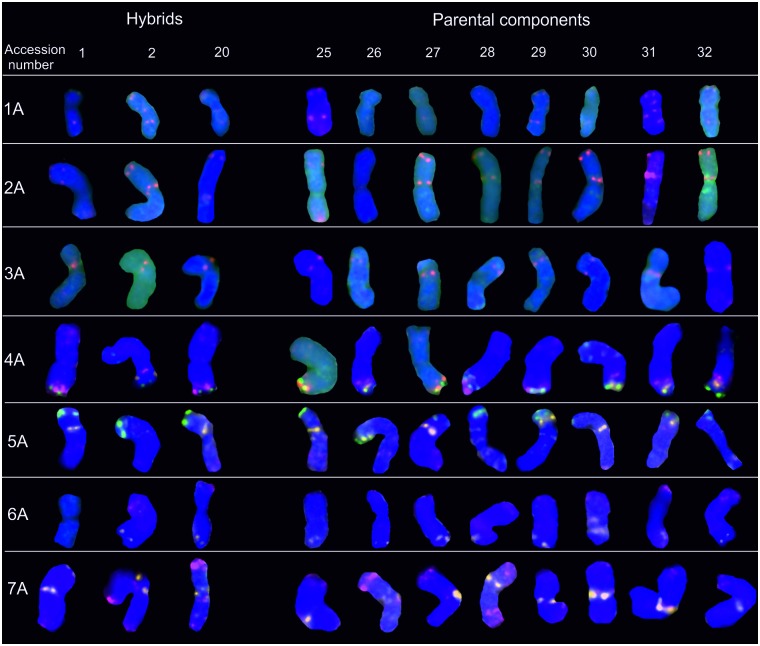
Karyograms of Torka x S10, Torka x S11, S10 x Kontesa hybrids and Torka, Kontesa, Zebra, S10-S14 parental components showing A-genome chromosomes after FISH with pTa-535 (red), pTa-86 (green) and pTa-713 (yellow) probes. Abbreviations: accession number 1- Torka x S10, 2- Torka x S11, 20- S10 x Kontesa, 25- Torka, 26- Kontesa, 27- Zebra, 28- S10, 29- S11, 30- S12, 31- S13, 32- S14.

The pTa-535 sequence produced the highest number of specific signals in A-genome chromosomes (Figs 2 and 3). The labeling patterns of the pTa-535 repetitive sequence were present in each A-genome chromosome. However, the intensity of the pTa-535 probe signals was rather low in all investigated wheat accessions. Hybridization patterns differed across chromosome types, but were similar in all tested wheat cultivars and their hybrids. Hybridization signals in subtelomeric regions of both arms of chromosomes 1A, 4AL and 5AL and in telomeric regions of 1AS, 6AS and both arms of chromosomes 2A and 7A were detected in hexaploid progenitors. A centromeric hybridization pattern was detected in two chromosomes (2A, 3A). Chromosomes carried 1 to 4 hybridization sites. Some hybrids were characterized by a lower number of repetitive sequences, which decreased signal intensity (Fig 3). Only several pTa-86 sites were identified in A-genome chromosomes. Signals were observed in the long arm of chromosome 4A and in the short arm of chromosome 5A, both in telomeric regions (Figs 2 and 3). The hybridization pattern of probe pTa-713 was detected in three A-genome chromosomes: 5AS, 6AL (telomeric region) and 7A (centromeric region) in most investigated accessions. Only the pTa-713 hybridization pattern was polymorphic—in chromosomes 4A and 6A. In chromosome 4A of *T*. *spelta* accession S10, the subtelomeric pTa-713 signal was not detected in the long arm of the chromosome (Fig 2). A comparison of hybrids revealed the polymorphic site of subtelomeric pTa-713 labeling in chromosome of 4A in Torka x S10 and S10 x Kontesa crosses (Figs 2 and 3). Probe pTa-713 did not produce a signal in the Torka x S10 accession in chromosome 6A (Figs 2 and 3).

### B-genome chromosomes

**Fig 4:**
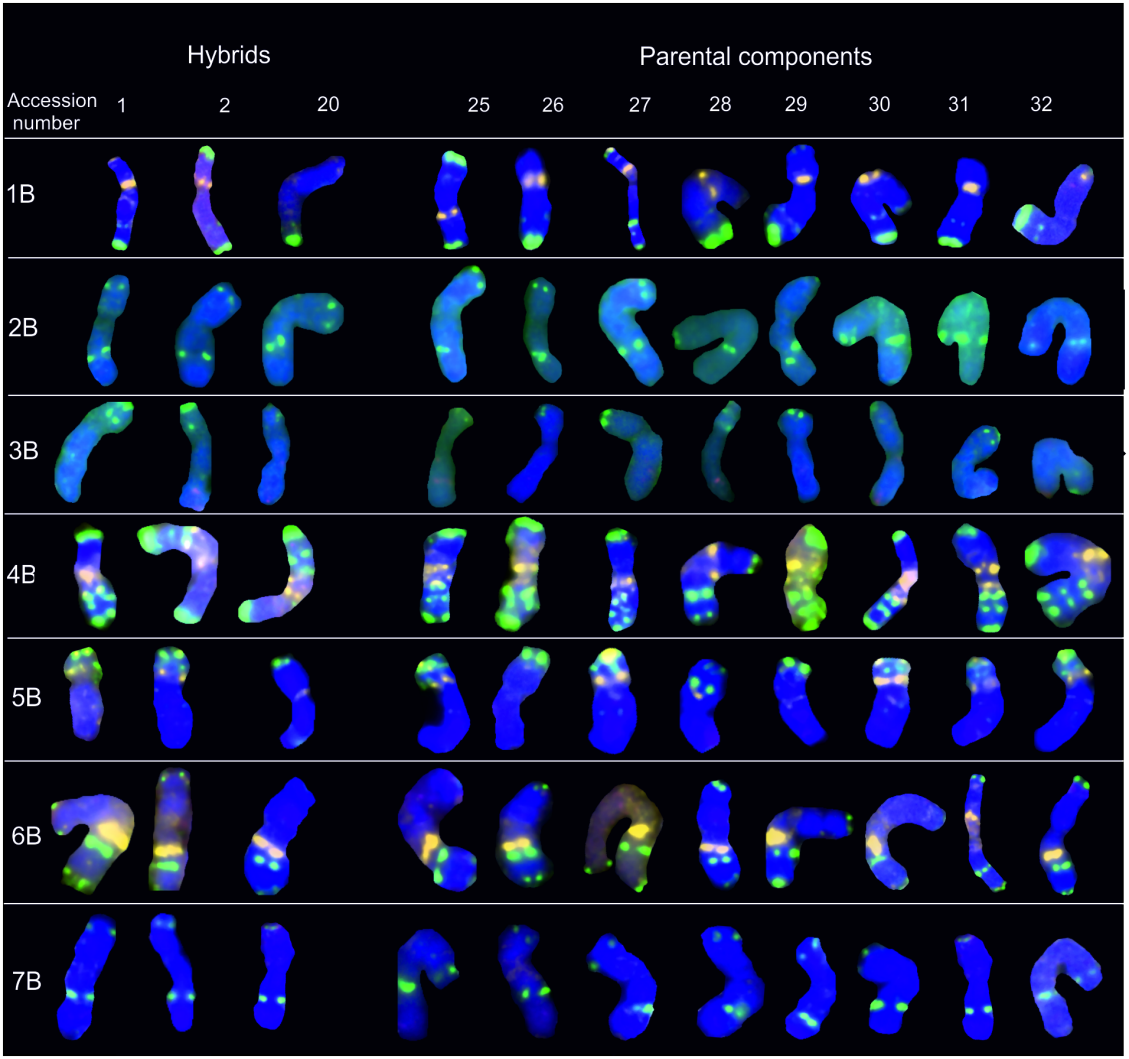
Karyograms of Torka x S10, Torka x S11, S10 x Kontesa hybrids and Torka, Kontesa, Zebra, S10-S14 parental components showing B-genome chromosomes after FISH with pTa-535 (red), pTa-86 (green) and pTa-713 (yellow) probes. Abbreviations: accession number 1- Torka x S10, 2- Torka x S11, 20- S10 x Kontesa, 25- Torka, 26- Kontesa, 27- Zebra, 28- S10, 29- S11, 30- S12, 31- S13, 32- S14.

Repetitive sequences pTa-535 were not present in the majority of B-genome chromosomes, and they generated weak and variable signals in the tested lines (Figs 2 and 4). pTa-535 labeling was present only in chromosomes 3B and 7B. Nonetheless, pTa-535 signal distribution facilitated chromosome identification. In all accessions, the most informative pTa-535 patterns were detected in chromosome 7B in the subtelomeric region of the short arm.

The pTa-86 hybridization pattern in B-genome chromosomes was strong and detectable in each chromosome of the analyzed accessions. Minor changes in signal intensity were observed between the examined common wheat cultivars and spelt lines and their hybrids (Fig 4). pTa-86 signals were detected mainly in telomeric and subtelomeric regions. Polymorphic sites of pTa-86 probe were detected in the short arms of chromosomes 1B and 6B in the telomeric regions. In Torka x S10 and S10 x Kontesa hybrids and S10 and S14 parental components, the pTa-86 labeling in 1B was replaced with pTa-713 probe signal (Fig 4). Another polymorphic hybridization pattern of pTa-86 was reported in 6B chromosome in telomeric region of short arm of chromosome. In accessions Torka x S11 (Fig 4), Torka x S12, Kontesa x S11, S10 x Kontesa (Fig 4), S11 x Torka and S12 x Torka, S11, S12, Torka signal was not observed.

The signal intensity of the pTa-713 probe differ in B-genome chromosomes. An intense signal generated by pTa-713 was detected in the centromeric region of chromosomes 1B and 4B and pericentromeric region of chromosome 6B. The pTa-713 signals in subtelomeric regions of chromosomes 5B was weaker but still strong. Weak, polymorphic pTa-713 signal was present in two parental components: S10 and S14 and two hybrids: Torka x S10 and S10 x Kontesa (Fig 4).

### D-genome chromosomes

**Fig 5:**
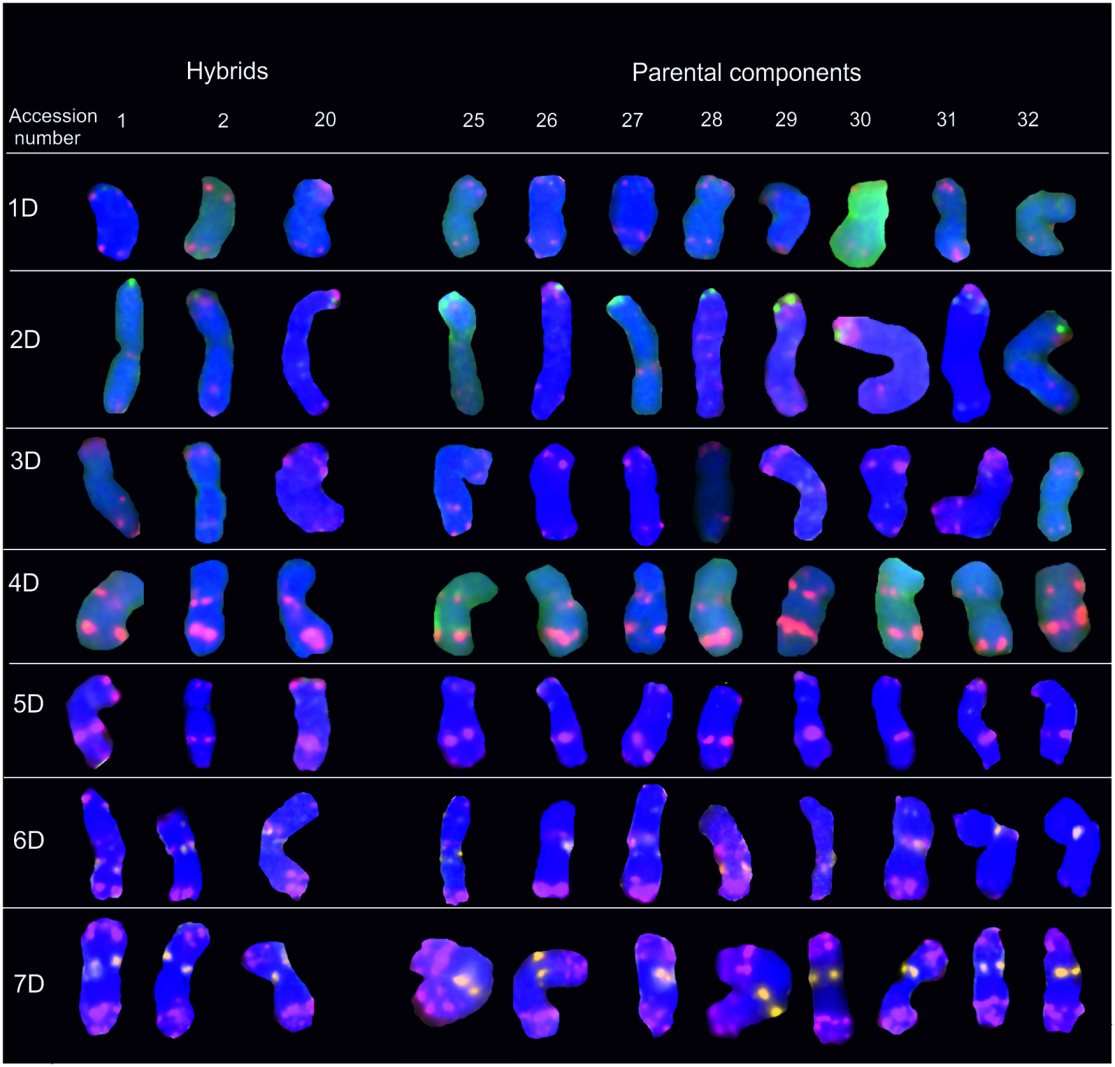
Karyograms of Torka x S10, Torka x S11, S10 x Kontesa hybrids and Torka, Kontesa, Zebra, S10-S14 parental components showing D-genome chromosomes after FISH with pTa-535 (red), pTa-86 (green) and pTa-713 (yellow) probes. Abbreviations: accession number 1- Torka x S10, 2- Torka x S11, 20- S10 x Kontesa, 25- Torka, 26- Kontesa, 27- Zebra, 28- S10, 29- S11, 30- S12, 31- S13, 32- S14.

In the FISH procedure, the pTa-535 probe produced strong and intense signals in D-genome chromosomes which were most informative in the group of the tested probes (Figs 2 and 5). Only pTa-535 tandem sequences were observed in D-genome chromosomes. pTa-535 labeling was present at various positions across entire chromosomes. Exceptionally high staining intensity was found in chromosome 4D (long arm, subtelomeric region) and in both arms of chromosome 7D (telomeric regions) in the analyzed accessions. The repetitive sequences pTa-86 were only present in chromosome 2D. The pTa-713 signal was detected only in the pericentromeric and centromeric regions of chromosomes 6D and 7D, respectively (Fig 5).

### 35S rDNA mapping

**Fig 6:**
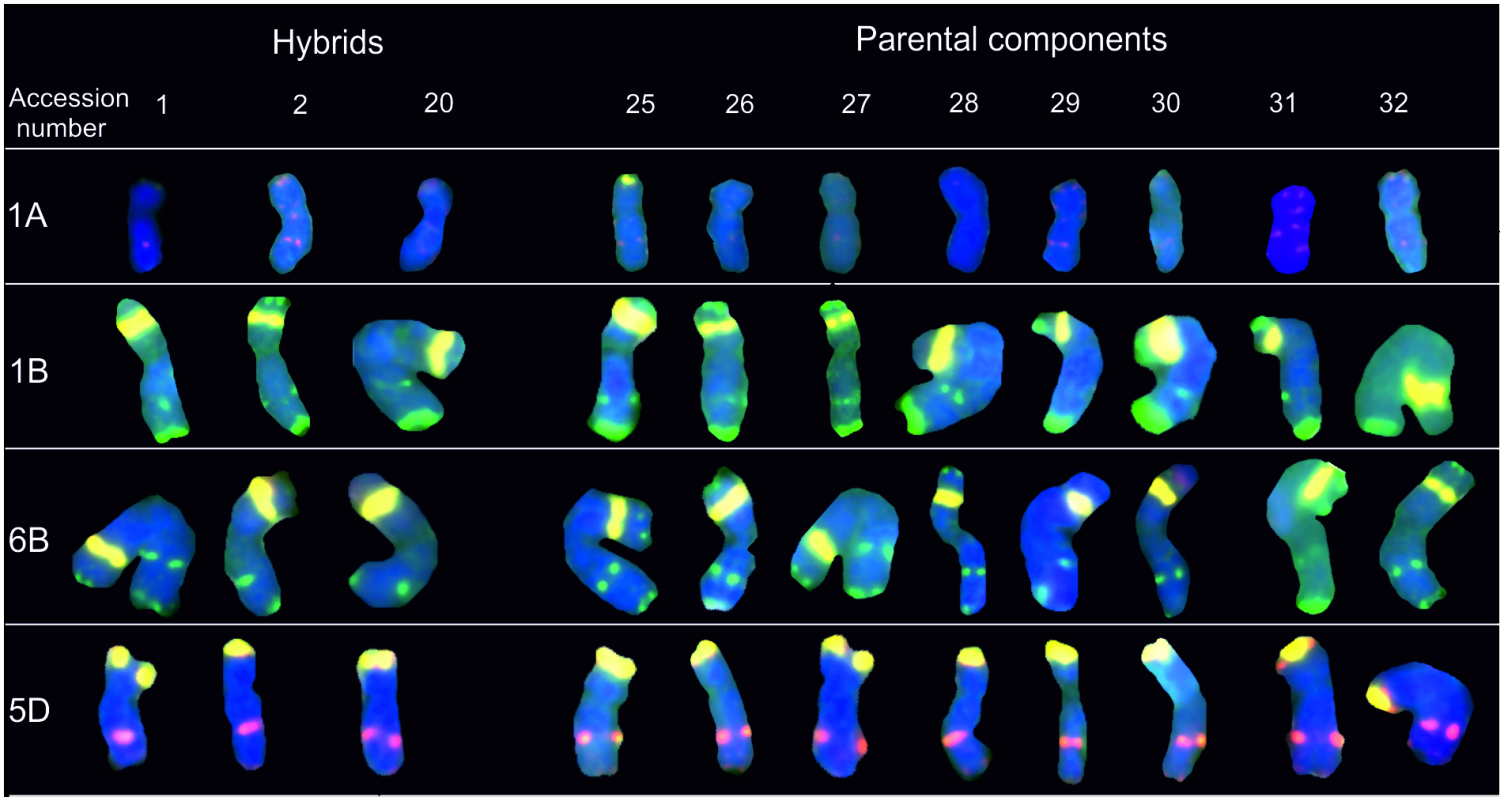
Karyograms of Torka x S10, Torka x S11, S10 x Kontesa hybrids and Torka, Kontesa, Zebra, S10-S14 parental components showing 1A, 1B, 6B and 5D chromosomes after FISH with pTa-535 (red), pTa-86 (green) and 35S rDNA (yellow) probes. Abbreviations: accession number 1- Torka x S10, 2- Torka x S11, 20- S10 x Kontesa, 25- Torka, 26- Kontesa, 27- Zebra, 28- S10, 29- S11, 30- S12, 31- S13, 32- S14.

The metaphases of wheat cultivars and their descendants had 3 or 4 pairs of chromosomes with 35S rDNA signals in a total of 42 chromosomes. Of these, 3 pairs of chromosomes (1B, 6B and 5D) always had the 35S rDNA hybridization pattern (Fig 6). An additional 35S rDNA signal was observed in chromosome 1A of *T*. *aestivum* cultivar Torka progenitors in the telomeric region of the short arm (Fig 6). Signal intensity was arranged in the following order: 1B>6B>5D>1A.

## Discussion

The analyzed *T*. *aestivum x T*. *spelta* and *T*. *spelta x T*. *aestivum* single-cross hybrids and their parental forms are allohexaploids (2n = 6x = 42). The structural similarity of chromosomes and their conjugation in hybrids produces BBAADD genomes [26]. Cytogenetic analyses involving the appropriate molecular techniques are increasingly used in breeding programs. Fluorescence *in situ* hybridization (FISH) is a cytogenetic technique that uses fluorescent probes to identify chromosomes according to their sequence [27]. None of the structural aberrations (translocations, inversions and deletions) were present in parental wheat cultivars and their hybrids.

A specific PCR reaction was initially performed to identify the 1BL/1RS translocation in the analyzed parental lines. Despite the fact that the 1BL/1RS translocation is widely used in breeding programs (in rye, the 1RS chromosome arm carries genes encoding resistance to rust (*Yr9*, *Sr31*, *Lr26*) and powdery mildew (*Pm8*)) [28], it was not detected in any of the parental spelt lines or common wheat cultivars. Negative PCR results for the 1BL/1RS translocation in all analyzed wheat lines was the confirmation of its absence in FISH results. The observed absence of the 1BL/1RS translocation was partially consistent with the findings of Kowalczyk et al. [29] who analyzed translocations in the short arm of rye (*Secale cereale* L.) chromosome 1RS onto common wheat. In the cited study, the 1BL/1RS translocation was absent in *T*. *aestivum* cv. Torka Our study made the first ever attempt to detect the 1BL/1RS translocation in common wheat cultivars (Kontesa and Zebra), self-bred lines of spelt and their crosses. This translocation is globally widespread in common wheat due to the presence of genes encoding resistance to selected diseases and pests. The 1BL/1RS translocation has been retained in many breeding programs [30] because it contributes to an increase in yield potential [31,32] and wheat growth dynamics under drought conditions [32–34]. It should also be noted that the 1BL/1RS translocation increases dough stickiness and decreases dough strength, traits that are not admissible in the grain of bread-making quality [35]. The absence of the 1BL/1RS translocation is a desirable trait which increases the bread-making quality and nutritional value of common wheat and spelt hybrids.

Karyotypes of investigated parental components of common wheat and spelt and their hybrids confirmed the BBAADD genome composition [36]. Repetitive sequences considerably influence genome evolution, and they can be used in analyses of genome diversity and phylogenetic reconstruction. Repetitive sequences also provide information about chromosomal rearrangements [37,38]. In higher plants, including members of the genus *Triticum*, physical maps are usually developed based on repetitive DNA sequences which are easier to identify and describe than low-copy genes [27,38,39]. Physical mapping of four repetitive sequences pTa-535, pTa-86 and pTa-713 and 35S rDNA in the chromosomes of common wheat cultivars, spelt lines and their hybrids revealed some polymorphic sites within analyzed parental components and their hybrids. Additionally, differences in repetitive sequences distribution have been detected between genomes of investigated wheats and standard cultivar for wheat cytogenetic research- *T*. *aestivum* cv. Chinese Spring [22]. Probes used in this study were enabled to distinguish wheat A-,B- and D-chromosomes (S3 Table). Moreover, these FISH markers captured the differences between investigated hybrids and their parental components. Few polymorphic sites in chromosomes were observed. Moreover, the use of pTa-535, pTa-86, pTa-713 and 35S rDNA probes allowed to trace the intensity of the signals. The presence of the cytoplasmic residues on chromosomes resulted in non-specific green background in certain wheat accessions (for example spelt accessions: 32 (Fig 3) or 30 (Fig 5)). However, it did not affect the signals reading. The observed signal strength and the number of pTa-535 repetitive sequences in A-genome chromosomes in hybrids and their progenitors were indicative of low polymorphism. A-genome chromosomes were characterized by decreased number of pTa-535 repetitive elements in comparison with D-genome chromosomes. In accessions with significant lower number of pTa-535, the signal was weak and (in some accessions) detected only in the red channel in the imaging program (Fig 3). In the A-genome chromosomes, pTa-86 labeling pattern was crucial for the identification of chromosomes 4A. A comparison of hybrids revealed the absence of subtelomeric pTa-713 labeling in chromosome 4A in Torka x S10 and S10 x Kontesa crosses (Figs 2 and 3). In both accessions, *T*. *spelta* breeding line S10 was one of the progenitors. It can be assumed that in these hybrids, one chromosome 4A was inherited from the S10 progenitor of *T*. *spelta*. The pTa-713 signals in the centromeric region of chromosomes 7A contributed to more precise identification of the studied hybrids, especially during the identification of chromosomes 7A and 7D. A comparison with the study of Komuro et al. [22] who analyzed the distribution of repetitive sequence signals in chromosomes of *T*. *aestivum* cv. Chinese Spring revealed some differences in repetitive elements distribution. In the long arms of chromosomes 3A and 4A of the analyzed lines, the pTa-535 signals were not observed in telomeric and subtelomeric regions, respectively. Probe pTa-713 was not detected in chromosomes 1A and 6A. However, it should be noted that the signals obtained from probe pTa-713 in chromosome 1A of *T*. *aestivum* cv. Chinese Spring was rather not intense [22], which suggests that in our study, the absence of the signals could be attributed to a small number of repetitive sequences in the chromosome.

In B-genome chromosomes, hybridization patterns of the analyzed probes were more diversified than in A-genome chromosomes (Fig 4). Differences in the intensity of the signals were observed especially in chromosomes 1B, 3B, 5B. The pTa-86 labeling pattern of chromosomes 4B was discriminative and crucial for its identification. The intensity and distribution of the pTa-86 signals in 4B were constant in all accessions. Kwiatek et al. [21] pointed out that 4B chromosomes had lower diversity than rest of the B-genome chromosomes. Polymorphic sites were observed in 1B and 6B chromosomes (Fig 4). Our findings are consistent with the previous study [40]- the telomeric region of the short arm of chromosome 6B is polymorphic, probably due to evolutionary changes. The pTa-713 probe was highly helpful in identifying chromosome 5B.

Our study also revealed other differences. In the analyzed accessions, the hybridization patterns of the tested probes differed from those reported by Komuro et al. [22] in the Chinese Spring cultivar. The pTa-86 signals were more intense in the long arm of chromosome 1B, and additional signals generated by pTa-713 (Torka x S10, S10 x Kontesa, S10 and S14) or pTa-86 (Torka x S11, Torka x S12, Kontesa x S11, S11 x Torka and S12 x Torka, S12, Torka) were detected in the short arm of chromosome 1B (Fig 4). The presence of additional pTa-713 and pTa-86 signals in hybrids where spelt was the paternal component suggests that polymorphism is determined by this paternal form. In Kontesa x S11 hybrids, an additional pTa-86 signals were not detected on the short arm of chromosome 1B of the Kontesa maternal component, but it was identified on the S11 paternal component. In S11 x Kontesa hybrids, where spelt line S11 was the maternal component and common wheat cultivar Kontesa was the paternal component, an additional pTa-86 labeling pattern was not observed. The above could imply that when an additional pTa-86 signals are detected in both parental lines, such as cultivar Torka and spelt lines S11 and S12, the signals will also be present in hybrids (Torka x S11, Torka x S12, S11 x Torka and S12 x Torka). The identity of maternal and parental components was not important in the above lines.

Intraspecific polymorphisms between common wheat cv. Chinese Spring and other cultivars of common wheat and spelt could have resulted from minor changes in the genome. Komuro et al. [22] compared repetitive sequence signals in cultivar Chinese Spring and also detected intraspecific polymorphisms in the analyzed accessions: an absence of a clear pTa-713 signals, a stronger pTa-535 signals in the telomeric region of the long arm of chromosome 3B, a single pTa-713 signal in chromosome 5B, different localization of the pTa-713 hybridization pattern (in the short rather than the long arm of the chromosome) and an absence of pTa-86 labeling in the telomeric region of 7BL. The observed hybridization patterns of the tested probes in parental components and their hybrids indicate that B-genome chromosomes are more diversified than A-, and D-genome chromosomes. Significant variations in B-genome chromosomes of wheat were also reported by Salina et al. [41,42] and Levy and Feldman [43].

The D-genome of wheat appears to be less susceptible to evolutionary changes, and it is characterized by low chromosome diversity [44,45]. In this study, polymorphic sites were not identified in these chromosomes (Fig 5). The distribution of pTa-535, pTa-86 and pTa-713 signals was stable and repeatable. The intensity of hybridization patterns was similar across the examined lines.

According to Komuro et al. [22] and Badaeva et al. [46], pTa-535 is a useful tool for identifying A- and D-genome chromosomes in common wheat. The labeling patterns in common wheat and spelt were highly similar, and they can be used to identify these chromosomes in common wheat x spelt hybrids and spelt x common wheat hybrids. The results of the analysis of pTa-535, pTa-86, pTa-713 and 35S rDNA hybridization patterns suggest the presence of a close relationship between common wheat and spelt. According to many authors, *T*. *spelta* was the first hexaploid wheat species whose random mutations gave rise to other wheat species, including *T*. *aestivum* [4]. The degree of polymorphism between different cultivars of these hexaploid wheats and their hybrids is rather low and confirms their close affinity. The identified variations are not associated with large-scale chromosome rearrangements [36]. Common wheat is an allohexaploid species that originated from a small number of interspecific and intergeneric hybridizations. Processes such as a genetic bottleneck and the founder effect are responsible for its low phenotypic and genotypic variation [47]. However, due to the absence of selection processes in the past, spelt is characterized by considerable genetic variability, and it could be a potential donor of desirable genes [48]. The above could explain why selected common wheat and spelt hybrids are characterized by polymorphic distribution of repetitive sequences in chromosomes. Numerous authors have suggested that the B-genome of wheat is the most diversified genome with the highest number of polymorphic markers in allohexaploid wheat [48–51]. Gill [52] found that B-genome chromosomes are characterized by more C-banding than A- and D-genome chromosomes. As anticipated, the distribution pattern of repetitive sequences in B-genome chromosomes exhibited the highest number of polymorphic sites between the analyzed lines relative to the labeling patterns of wheat cultivar Chinese Spring [22]. The D-genome may be less diversified because allohexaploid wheat originated around 8500–9000 BC, and it is a source of repetitive sequence polymorphisms. The polymorphisms observed in common wheat and spelt hybrids carry important information for wheat breeders. The results of our study are also a valuable source of knowledge about genome organization and diversification in common wheat, spelt and their hybrids. The relevant information is essential for common wheat breeders, and it can contribute to breeding programs aimed at biodiversity preservation.

## Supporting information

S1 Table Description of the accessions used in this study. (PDF)

S2 Table Primers sequences and PCR conditions for wheat repetitive sequences amplification. (PDF)

S3 Table The pTa-535, pTa-86, pTa-713 and 35S rDNA repetitive sequences distribution on chromosomes. (PDF)
