# Correction: Chromosomal distribution of pTa-535, pTa-86, pTa-713, 35S rDNA repetitive sequences in interspecific hexaploid hybrids of common wheat (*Triticum aestivum* L.) and spelt (*Triticum spelta* L.)

**DOI:** 10.1371/journal.pone.0203162

**Published:** 2018-08-23

**Authors:** Klaudia Goriewa-Duba, Adrian Duba, Michał Kwiatek, Halina Wiśniewska, Urszula Wachowska, Marian Wiwart

There is an error in the affiliation of author Halina Wiśniewska. The affiliation should be: Institute of Plant Genetics, Polish Academy of Sciences, Poznań, Wielkopolskie Voivodeship, Poland.
