# Supplementary material for: Chromosomal distribution of pTa-535, pTa-86, pTa-713, 35S rDNA repetitive sequences in interspecific hexaploid hybrids of common wheat (Triticum aestivum L.) and spelt (Triticum spelta L.)
Source: PLoS One. 2018 Feb 15;13(2):e0192862. doi: 10.1371/journal.pone.0192862 (PMC5813972; doi:10.1371/journal.pone.0192862)
Supplement: S1 Table — (PDF) [file pone.0192862.s001.pdf]

Table 1. Description of the accessions used in this study

Accession no	Hybrids	Origin
**1**	TORKA x S10	Line of own breeding, University of Warmia and Mazury
**2**	TORKA x S11	Line of own breeding, University of Warmia and Mazury
**3**	TORKA x S12	Line of own breeding, University of Warmia and Mazury
**4**	TORKA x S14	Line of own breeding, University of Warmia and Mazury
**5**	KONTESA x S10	Line of own breeding, University of Warmia and Mazury
**6**	KONTESA x S11	Line of own breeding, University of Warmia and Mazury
**7**	KONTESA x S12	Line of own breeding, University of Warmia and Mazury
**8**	KONTESA x S13	Line of own breeding, University of Warmia and Mazury
**9**	KONTESA x S14	Line of own breeding, University of Warmia and Mazury
**10**	ZEBRA x S10	Line of own breeding, University of Warmia and Mazury
**11**	ZEBRA x S11	Line of own breeding, University of Warmia and Mazury
**12**	ZEBRA x S12	Line of own breeding, University of Warmia and Mazury
**13**	ZEBRA x S13	Line of own breeding, University of Warmia and Mazury
**14**	ZEBRA x S14	Line of own breeding, University of Warmia and Mazury
**15**	S10 x TORKA	Line of own breeding, University of Warmia and Mazury
**16**	S11 x TORKA	Line of own breeding, University of Warmia and Mazury
**17**	S12 x TORKA	Line of own breeding, University of Warmia and Mazury
**18**	S13 x TORKA	Line of own breeding, University of Warmia and Mazury
**19**	S14 x TORKA	Line of own breeding, University of Warmia and Mazury
**20**	S10 x KONTESA	Line of own breeding, University of Warmia and Mazury
**21**	S11 x KONTESA	Line of own breeding, University of Warmia and Mazury
**22**	S12 x KONTESA	Line of own breeding, University of Warmia and Mazury
**23**	S13 x KONTESA	Line of own breeding, University of Warmia and Mazury
**24**	S14 x KONTESA	Line of own breeding, University of Warmia and Mazury
	**Parental components**	
**25**	*Triticum aestivum* cv. Torka	Material obtained from seed company (own reproduction), University of Warmia and Mazury
**26**	*Triticum aestivum* cv. Kontesa	Material obtained from seed company (own reproduction), University of Warmia and Mazury
**27**	*Triticum aestivum* cv. Zebra	Material obtained from seed company (own reproduction), University of Warmia and Mazury
**28**	S10	Spelt line of own breeding, University of Warmia and Mazury
**29**	S11	Spelt line of own breeding, University of Warmia and Mazury
**30**	S12	Spelt line of own breeding, University of Warmia and Mazury
**31**	S13	Spelt line of own breeding, University of Warmia and Mazury
**32**	S14	Spelt line of own breeding, University of Warmia and Mazury
