# Supplementary material for: Chromosomal distribution of pTa-535, pTa-86, pTa-713, 35S rDNA repetitive sequences in interspecific hexaploid hybrids of common wheat (Triticum aestivum L.) and spelt (Triticum spelta L.)
Source: PLoS One. 2018 Feb 15;13(2):e0192862. doi: 10.1371/journal.pone.0192862 (PMC5813972; doi:10.1371/journal.pone.0192862)
Supplement: S2 Table — (PDF) [file pone.0192862.s002.pdf]

Table 2. Primers sequences and PCR conditions for wheat repetitive sequences amplification

Clone	NCBI GenBank accession number	Website address	Primer sequence (5'->3')	Annealing temperature (°C)	Product length (bp)
**pTa-535**	KC290894.1	https://www.ncbi.nlm.nih.gov/nuccore/KC290894.1	GCATAGCATGTGCGAAAGAG	58	101
			TCGTCCGAAACCCTGATAC		
**pTa-86**	KC290896.1	https://www.ncbi.nlm.nih.gov/nuccore/KC290896.1	ACGATTGACCAATCTCGGGG	58.5	531
			ACCGACCCAAATTACGAGAGT		
**pTa-713**	KC290900.1	https://www.ncbi.nlm.nih.gov/nuccore/KC290900.1	GGGGCGGACGTCGTTG	59	337
			CCGTAAGATAGACAGGGTGGG		
**pTa-k374 (35S rDNA)**	KC290907	https://www.ncbi.nlm.nih.gov/nuccore/KC290907	TTTCAACCAAGCGCGATGAC	59	690
			ATCAGCGGGGAAAGAAGACC		
