# Supplementary material for: Chromosomal distribution of pTa-535, pTa-86, pTa-713, 35S rDNA repetitive sequences in interspecific hexaploid hybrids of common wheat (Triticum aestivum L.) and spelt (Triticum spelta L.)
Source: PLoS One. 2018 Feb 15;13(2):e0192862. doi: 10.1371/journal.pone.0192862 (PMC5813972; doi:10.1371/journal.pone.0192862)
Supplement: S3 Table — (PDF) [file pone.0192862.s003.pdf]

Table 3. The pTa-535, pTa-86, pTa-713 and 35S rDNA repetitive sequences distribution on chromosomes

Chromosome	Arm	pTa-535	pTa-86	pTa-713	pTa-k374 (35S rDNA)
**1A**	S	T, ST, C	-	-	- or T
	L	ST,C	-	-	-
**2A**	S	T, C	-	-	-
	L	T,C	-	-	-
**3A**	S	C	-	-	-
	L	C	-	-	-
**4A**	S	-	-	-	-
	L	T or ST	T or ST	ST	-
**5A**	S	-	T	C	-
	L	ST	-	C	-
**6A**	S	T	-	-	-
	L	-	-	- or T	-
**7A**	S	T, ST	-	C	-
	L	T	-	C	-
**1B**	S	-	- or T	- or T, C	ST
	L	-	T, ST	C	-
**2B**	S	-	T	-	-
	L	-	ST	-	-
**3B**	S	-	T, ST	-	-
	L	T, ST	-	-	-
**4B**	S	-	T	C	-
	L	-	T, ST	C	-
**5B**	S	-	T	C	-
	L	-	-	C	-
**6B**	S	-	- or T	C	ST
	L	-	T, ST	C	-
**7B**	S	ST	ST	-	-
	L	-	ST	-	-
**1D**	S	T	-	-	-
	L	T	-	-	-
**2D**	S	T, ST, C	T	-	-
	L	T, ST, C	-	-	-
**3D**	S	T, ST	-	-	-
	L	T	-	-	-
**4D**	S	C	-	-	-
	L	ST,C	-	-	-
**5D**	S	T	-	-	T
	L	ST	-	-	-
**6D**	S	T,ST	-	C	-
	L	T	-	C	-
**7D**	S	T	-	C	-
	L	T	-	C	-

Abbreviations: S- short arm, L- long arm, T- telomeric region, ST- subtelomeric region, C- centromeric region, - absence of the signal
